# Anti-microbial and anti-mycotoxigenic activities of endophytic *Alternaria alternata* isolated from *Catharanthus roseus* (L.) G. Don.: molecular characterisation and bioactive compound isolation

**DOI:** 10.1080/21501203.2018.1541933

**Published:** 2018-11-05

**Authors:** T. N. Sudharshana, H. N. Venkatesh, Borah Nayana, K. Manjunath, D. C. Mohana

**Affiliations:** Department of Microbiology and Biotechnology, Bangalore University, Bengaluru, India

**Keywords:** Endophytic *Alternaria alternata*, ethyl acetate extract, *p*-Coumaric acid, anti-microbial activity, aflatoxin B_1_, fumonisin B_1_

## Abstract

The present study investigated the anti-microbial and anti-mycotoxigenic activities of the ethyl acetate extract (EA) and a bioactive compound obtained from an endophytic fungus *Alternaria alternata* isolated from *Catharanthus roseus* leaves. *A. alternata* was identified using PCR-based 5.8S rDNA sequencing. The EA and bioactive compound, *p*-Coumaric acid (PC), showed concentration-dependent broad-spectrum anti-microbial activity against the tested bacteria, yeast, and fungi with MICs ranging from 7.8 to 250 µg/mL. The *in vitro* production of aflatoxin B_1_ (AFB_1_) from *Aspergillus flavus* and fumonisin B_1_ (FB_1_) from *Fusarium verticillioides* was completely inhibited by EA and PC at 400 µg/mL. The synthesis of the membrane-bound ergosterol from *A. flavus* and *F. verticillioides* was strongly inhibited by PC at 200 µg/mL. The EA and PC were found to show significant anti-microbial and anti-mycotoxigenic activities, hence, they could be explored as protective agents for preventing microbial deterioration and mycotoxins accumulation in food and feedstuffs during pre- and post-harvest and storage.

## Introduction

The xerophilic filamentous fungi belong to the genera *Aspergillus, Fusarium* and *Penicillium*. They are predominantly associated with food- and feed stuffs during pre- and post-harvest and storage, and make them unfit for human consumption by destroying the nutritive values due to the synthesis of mycotoxins (Lutfullah et al. 2012; Abhishek et al. 2015). The most important groups of mycotoxins that are a major threat to human and animal health, and occur quite often in foodstuffs are aflatoxin B_1_ (AFB_1_) and fumonisin B_1_ (FB_1_) produced by toxigenic strains of *A. flavus* and *F. verticillioides*, respectively (Garcia et al. 2012; Mohana et al. 2016). In addition, the increasing multidrug-resistant (MDR) food-borne human pathogens, particularly the strains of *Staphylococcus aureus, Escherichia coli, Salmonella typhimurium, Klebsiella pneumoniae* and *Candida albicans* are the greatest and most urgent global risk (Xu et al. 2014). Nowadays, many antibiotics and pesticides of synthetic origin are being widely used to manage problems associated with MDR microbes, moulds and mycotoxins, but their continuous and indiscriminate use has intensified the development of MDR micro-organisms and resulted in many ecological problems (Calbo et al. 2011). In this scenario, there is an urgent need to search alternative strategies that are eco-friendly and effective.

Endophytic fungi are omnipresent plant symbionts, reside inter- or intra-cellularly inside a host plant without causing any conspicuous infection (Guo et al. 2000; Ayob et al. 2016). The endophytic fungi indirectly benefit plant growth by producing many vital compounds including bioactive secondary metabolites (Kaul et al. 2012; Lou et al. 2013). Even though, many valuable bioactive compounds have been successfully identified from endophytic fungi (Yu et al. 2010; Bhagat et al. 2016), only a few investigations have been focused on the exploration of endophytic fungi and their bioactive molecules in food, agriculture and pharmaceuticals (Ezra et al. 2004; Yu et al. 2010; Kaul et al. 2012; Palem et al. 2015). In this backdrop, the present investigation was undertaken to evaluate the anti-microbial and anti-mycotoxigenic properties of *A. alternata*, an endophytic fungus isolated from the leaves of *Catharanthus roseus* (L.) G. Don., which is a well-known medicinal plant belonging to the family Apocynaceae.

## Material and methods

### Isolation of endophytic fungi

Endophytic fungi were isolated from the leaves of *C. roseus* following the procedure of Ezra et al. (2004). Briefly, fresh plant samples of *C. roseus* were collected from Charaka-Sushruta Vana, Bengaluru (India), and authenticated by Dr. Seetharam, Professor, Department of Biological Sciences, Bangalore University, Bengaluru (India). The authenticated voucher specimen (BUB/MB-BT/DCM/2016/08) was deposited in the Department of Microbiology and Biotechnology, Bangalore University, Bengaluru (India).

The collected leaf samples were cut into small pieces (0.5 × 0.5 cm), surface sterilised successively with 1% sodium hypochlorite and 90% ethanol for 3 min, and placed in potato-dextrose-agar (PDA) plates. The plates were incubated up to 7 days at 28 ± 2°C. The hyphal tips emerging out of the leaf samples were picked up using stereomicroscope (SZ-PT, Olympus Corporation, Tokyo, Japan) and sub-cultured on fresh PDA. The pure culture of the isolate DCM-EFS-11 showing the highest anti-microbial activity was selected for identification using the 5.8S rDNA sequence analysis followed by a binocular compound microscopic (CX41RF, Olympus Corporation, Tokyo, Japan) images analysis (Nagamani et al. 2006; Watanabe et al. 2010).

### 5.8S rDNA sequence analysis of the isolate DCM-EFS-11

The 5.8S rDNA sequence of the isolate DCM-EFS-11 was analysed following the procedure of Mohana et al. (2016). Briefly, the genomic DNA from fresh culture DCM-EFS-11 was isolated using genomic DNA isolation kit procured from Aristogene Bioscience Pvt. Ltd. (Bengaluru, India). The isolated DNA was amplified using a PCR machine (Q cycler, CM 6050, Quanta Biotech, England). The fungal-specific internal transcribed spacer (ITS) regions (5´TGATCCTTCYGCAGGTTCAC3´ and 5´ACCTGGTTGATCCTGCCAG3´) were used as forward and backward primers to amplify a fragment within the gene coding for 5.8S rRNA. The PCR products were analysed by electrophoresis and the separated bands were subjected to genome sequencing at Eurofins Genomics Pvt. Ltd., Bengaluru (India). The collected 5.8S rDNA sequences were searched against GenBank using NCBI-BLAST search tool and a phylogenetic tree was constructed based on the similarities between the related taxa. The isolate fungal strain DCM-EFS-11 was identified using a phylogenetic tree followed by microscopic image analysis and the base sequence of the identified fungal strain was deposited in NCBI-GenBank (accession no. MG551266).

### Ethyl acetate extraction and bioactive compound isolation from the culture filtrates of endophytic A. alternata

Ethyl acetate extract was prepared from the culture filtrate of *A. alternata* following the procedure of Srivastava and Anandrao (2015). Briefly, *A. alternata* was grown in 500 mL PDB up to 15 days at 28°C and filtered through a Whatman grade 1 filter paper. The collected filtrate (100 mL) was extracted with the same amount of ethyl acetate using a separatory funnel, and the collected ethyl acetate fraction was concentrated to dryness using a vacuo evaporator (Lyoquest-85, Telstar Technologies, S.L. Terrassa, Spain). The dried crude ethyl acetate extract (EA) was collected (45 mg) and then subjected to bioactivity evaluation followed by compound isolation using column chromatography as described by Mohana et al. (2010). Briefly, a silica gel chromatographic column (mesh size 60–120, SRL, India) loaded with EA was eluted sequentially with the mixtures of CHCl_3_ and MeOH (10:0, 7.5:2.5, 1:1, 2.5:7.5, 0:10, v/v). The collected chromatogram fractions were allowed to dry and all the fractions were subjected for anti-microbial activity evaluation. The fraction showing the highest activity was selected and further purified using TLC where CHCl_3_: MeOH (9:1) served as the mobile phase. The TLC bands were scraped out, dissolved individually in MeOH, filtered using Whatman grade 1 filter paper, and allowed to dry. The pure crystals (08 mg) obtained from the third band were collected and subjected to anti-microbial evaluation followed by ESI-MS and FT-IR spectral analysis. The bioactive compound was identified by comparing the spectral data with the published literature.

## Evaluation of anti-microbial activities of EA and PC

### Microbes tested

In order to test the anti-microbial activity of EA and PC, following microbes were used: seven bacterial species, viz., *Escherichia coli, Klebsiella pneumoniae, Proteus vulgaris, Pseudomonas aeruginosa, Salmonella typhimurium, Staphylococcus aureus* and *Streptococcus faecalis*; two yeast species, viz., *Candida albicans* and *Cryptococcus neoformans*; and 15 species of fungi, viz., *Alternaria brassicicola, Alternaria geophila, Aspergillus flavus* (aflatoxigenic strain), *Aspergillus fumigatus, Aspergillus ochraceus, Aspergillus tamarii, Aspergillus terreus, Curvularia tetramera, Fusarium oxysporum, Fusarium lateritium, Fusarium equiseti, Fusarium udum, Fusarium verticillioides* (fumonisinogenic strain), *Penicillium citrinum* and *Penicillium expansum*. The collection centres and sources of these isolates have been described earlier (Thippeswamy et al. 2014; Abhishek et al. 2015). The cultures, grown for 24 h, 48 h and 7 days for bacteria, yeast and fungi, respectively, were used for the assay.

### Evaluation of anti-bacterial and anti-yeast activities

The disc diffusion method was employed for the evaluation of anti-bacterial and anti-yeast activities of EA and PC against seven bacterial and two yeast species (Hajji et al. 2010). Briefly, the discs (6 mm in diameter) impregnated with EA (150 µg/disc) and PC (100 µg/disc) were placed on the pre-inoculated MHA (10^8^ CFU/mL of bacteria) and MGYPA (10^6^ CFU/mL of yeast) plates and incubated at 37°C for 24 h for bacteria and 48 h for yeast. The disc impregnated with the same amount of DMSO served as a negative control and the antibiotics erythromycin (15 mcg) and itraconazole (10 mcg) served as positive control for bacterial and yeast species, respectively. The anti-microbial activities of EA and PC were determined by measuring the zone of inhibition (ZOI) around the discs.

## Evaluation of anti-fungal activity

The poisoned food technique was employed for the evaluation of anti-fungal activities of EA and PC against 15 seed-borne fungal species following the procedure of Mohana et al. (2010). Briefly, mycelial discs (5 mm in diameter) of the test fungi were placed on SDA impregnated with EA (150 µg/mL) and PC (100 µg/mL), and incubated at 28 ± 2°C for 7 days. The SDA impregnated with the same amount of DMSO served as a negative control and zinc ethylene bisthiocarbamate (Indofil Z-78) (2 mg/mL) served as a positive control. The anti-fungal activities of EA and PC were measured by calculating the percentage of mycelial growth inhibition (%MI) using the formula given below.

$$\%MI=\left(C-T/C\right)\times 100$$

where *C* is the diameter of mycelial growth in control plate and *T* is the diameter of mycelial growth in a treated plate.

### Determination of MICs of EA and PC against microbes

The broth dilution technique was employed for the determination of MICs of EA and PC (Hajji et al. 2010). Briefly, 15 µL of bacteria (10^8^ CFU/mL), yeast (10^6^ CFU/mL), and fungi (10^4^ CFU/mL) were independently inoculated into a microtiter plate containing 200 µL of MHB, MGYPB, and SDB impregnated with twofold dilution of EA and PC (3.9 µg/mL–2 mg/mL), respectively, and incubated for a specific period and at temperature as explained above. After incubation, 50 µL of iodo-nitro-tetrazolium chloride (INT 2 mg/mL) was added to each well and incubated further for 30 min. The pale yellow-coloured INT was reduced to pink indicating the presence of viable cells and no change in colour indicated the inhibition of microbial growth. The lowest concentration at which the colour remained unchanged was considered as MIC. DMSO served as a negative control.

## Evaluation of anti-mycotoxigenic activities of EA and PC

The *in vitro* anti-aflatoxin B_1_ and anti-fumonisin B_1_ activities of EA and PC were determined following the procedure of Abhishek et al. (2015). Briefly, 100 µL of spore suspension (10^4^ CFU/mL) of a toxigenic strain (*A. flavus*) was inoculated into the SMKYB, and *F. verticillioides* was inoculated into the SDA containing the requisite amount of EA and PC (50, 100, 200 and 400 µg/mL), and incubated at 28°C for 10 days. The medium devoid of EA and PC served as control. After incubation, AFB_1_ was extracted from the culture filtrate of *A. flavus* by adding an equal volume of CHCl_3_ (Shukla et al. 2008), and FB_1_ was extracted from a culture of *F. verticillioides* by adding an equal amount of acetonitrile-water mixture (1:1, v/v) (Baily et al. 2005).

The extracted AFB_1_ was spotted on the TLC plate adjacent to AFB_1_ standard (Sigma, Germany), then eluted using CHCl_3_-acetone (96:4, v/v) as the mobile phase and observed under ultraviolet light at 365 nm (UV-cabinet, Labline, India). AFB_1_ was estimated qualitatively by visual comparison of the fluorescence intensity of the samples with standard spots, and quantitatively by measuring the light absorbance of the samples using a spectrophotometer at 600 nm wavelengths (UV-1800, Shimadzu, Japan). The amount of AFB_1_ content was calculated by the formula given below.

$$AFB_{1}\left(\mu g/L\right)=\left[D\times M/E\times L\right]1000$$

where *D* is absorbance, *M* is the molecular weight of AFB_1_ (312), *E* is the molar extinction coefficient of AFB_1_ (21,800) and *L* is the path length (1 cm).

The extracted FB_1_ was spotted on the TLC plate adjacent to standard FB_1_ (Sigma, Germany) and eluted using a mixture of butanol-acetic acid-water (20:10:10, v/v/v) as the mobile phase. The solution of *p*-anisaldehyde in methanol-acetic acid-H_2_SO_4_ (85:10:0.5, v/v/v) (0.5%) was sprayed on the chromatogram and incubated at 110°C for 10 min. The amount of FB_1_ on chromatogram was estimated by comparing the band’s intensity with standard spots using a spectrophoto-densitometer at 600 nm wavelengths (BioRad, Universal Hood II 720BR/02170, USA).

The *in vivo* efficacy of EA and PC on inhibition of AFB_1_ and FB_1_ production was estimated using viable maize seed samples following the procedure of Bailly et al. (2005). Briefly, 100 µL spore suspension of toxigenic strains of *A. flavus* and *F. verticillioides* (10^4^ CFU/mL) was inoculated separately into the maize samples treated with different amount of EA and PC (50, 100, 200 and 400 µg/g). The maize seeds impregnated with EA and PC without spore inoculum of *A. flavus* and *F. verticillioides* were used for natural mycoflora analysis and seedling-vigour estimation following the procedure of ISTA (2006). The maize seeds without EA and PC served as control. Both treated and control maize samples were stored in plastic containers (200 g pack*^–^*^1^) separately up to 15 days at 25 ± 2°C. The water activity (*a_w_*) was adjusted to 0.95 by adding sterile distilled water (Mohana et al. 2014). After 15 days, all samples were powdered separately, *A. flavus* treated samples were used for AFB_1_ extraction followed by quantification, whereas *F. verticillioides*-treated maize samples were used for FB_1_ extraction followed by quantification using the same procedure as explained above in *in vitro* studies.

## Mode of anti-fungal action of PC against ergosterol synthesis in *A. flavus* and *F. verticillioides*

The mode of action of PC on the inhibition of ergosterol synthesis from the toxigenic strains of *A. flavus* and *F. verticillioides* was determined following the procedure of Tian et al. (2012). Briefly, 50 µL of spore suspension of the toxigenic strains of *A. flavus* (10^4^ spores/mL) was separately inoculated with SDB containing desired concentrations of PC (50, 100, 150 and 200 µg/mL) and incubated at 28 ± 2°C for 4 days. After incubation, the mycelial mat was collected and subjected to ergosterol extraction by adding a solution of alcoholic potassium hydroxide (7.5:2.5 v/v) and *n*-heptane. The extracted ergosterol was analysed by scanning between 230 and 300 nm in a spectrophotometer. The SDB without PC served as a positive control and SDB with only PC (without *A. flavus* and *F. verticillioides*) served as a blank. A characteristic curve at 230 and 282 nm indicates the presence of ergosterol. The percent ergosterol content was calculated using the formula given below.

$$\begin{array}{rl} & \%ergosterol\;+\;\%24\left(28\right)\;-\;dehydroergosterol\;=\\ & \;\;\;\;\;\;\;\;\;\;\;\;\;\;\;\;\;\;\;\;\;\;\;\;\;\;\;\;\;\;\;\;\;\;\;\;\;\;\;\;\;\;\;\;\;\;\;\;\left(A_{282}/290\right)/pelletweight, \end{array}$$

$$\begin{array}{rl} & \%24\left(28\right)\;-\;dehydroergosterol=\\ & \;\;\;\;\;\;\;\;\;\;\;\;\;\;\;\;\;\;\;\;\;\;\;\;\;\;\;\;\;\;\;\;\;\;\;\;\;\;\;\;\;\;\;\;\;\left(A_{230}/518\right)/pelletweight, \end{array}$$

$$\begin{array}{rl} & \%ergosterol\;=\;\left(\%ergosterol\;+\;\%24\left(28\right)\right.\\ & \left.\;\;\;\;\;\;\;\;\;\;\;\;\;\;\;\;\;\;\;\;\;\;\;\;\;\;\;\;\;-\;dehydroergosterol\right)\\ & \;\;\;\;\;\;\;\;\;\;\;\;\;\;\;\;\;\;\;\;\;\;\;\;\;\;\;\;\;\;\;\;\;\;\;\;-\;\%24\left(28\right)dehydroergosterol \end{array}$$

where 290 and 518 are the *E* values (in percentages per centimetre) of crystalline ergosterol and 24(28)-dehydroergosterol, respectively, and pellet weight is the net wet weight (g).

## Results

Among the 14 different endophytic fungi isolated from the leaf of *C. roseus*, an isolate DCM-EFS-11 showing the highest anti-microbial activity, was selected for 5.8S rDNA sequence analysis for identification. The sequence analysis of the isolate showed 99.0% similarity with *A. alternata* and 97–98% similarity with other *Alternaria* spp. The sequence analysis along with the microscopic image analysis identified the isolate to be *A. alternata*. The complete 5.8S rDNA nucleotide sequence of *A. alternata* was submitted to the NCBI GenBank (India), and an accession number (MG551266) was given.

**Figure 1.**
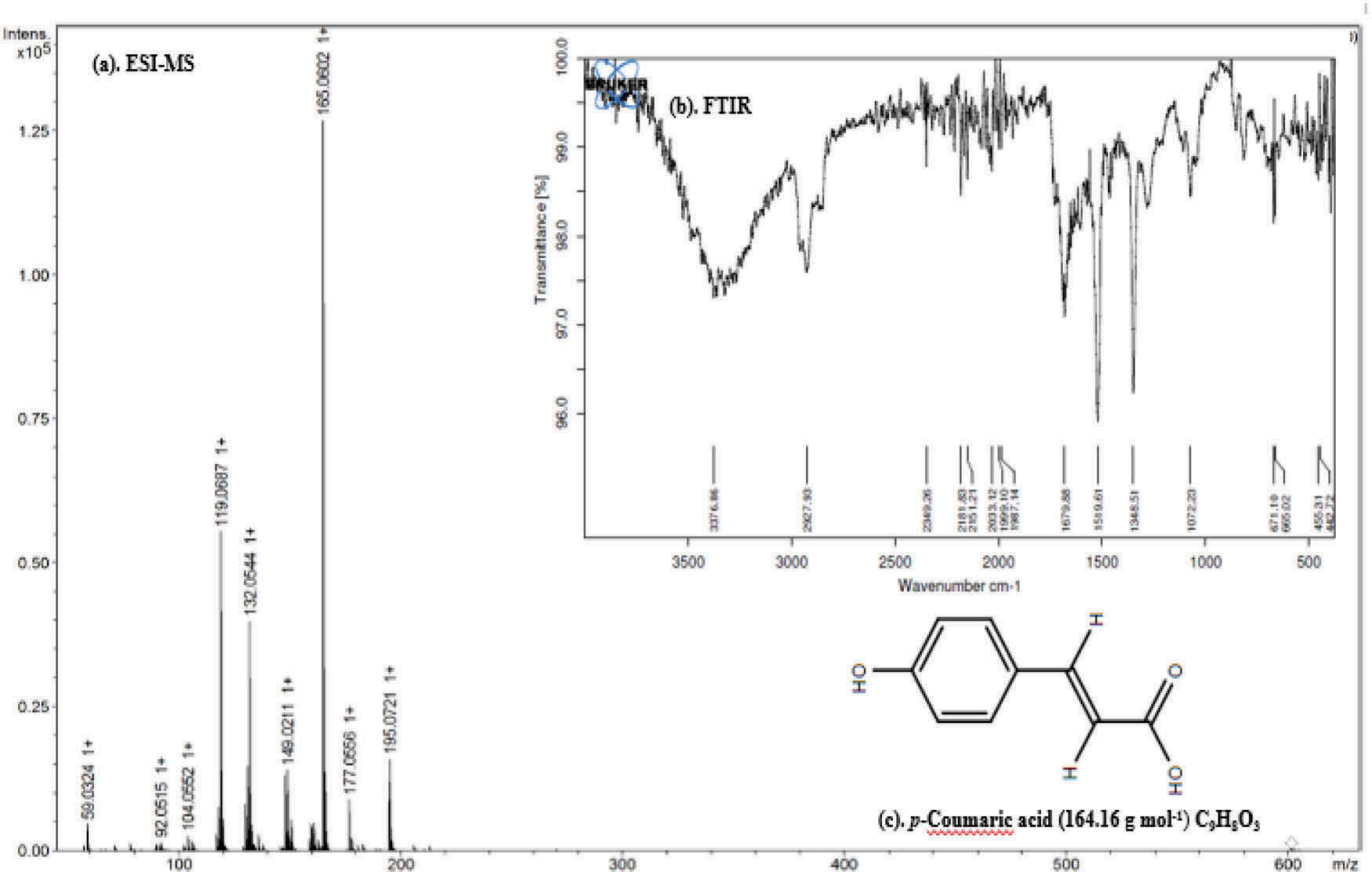
ESI-MS spectra (a), FT-IR spectra (b) and chemical structure of the p-Coumaric acid.

In the column chromatography separation, a total of 21 fractions (10 mL per fraction) were collected from EA, of which, eleventh fraction showed anti-microbial activity. Thus it was selected for further purification of the bioactive compound using the TLC system. In the TLC separation, a total of six bands were eluted with the *R_f_* values of 0.22, 0.30, 0.45, 0.63, 0.75 and 0.90. Among them, the third band with an *R_f_* value of 0.45 and showing anti-microbial activity was collected from the TLC, and subjected to ESI-MS and FT-IR analysis for identification. In the positive mode ESI-MS analysis, the isolated bioactive compound showed a molecular weight of 165.05 [M + H]^+^ (Figure 1). In the FT-IR spectra, the broad signals at 3376.86 (OH stretching) and 2927.93 (C-H aromatic + (CH)_C=C_), sharp peak at 1679.88 (-CC aromatic), 1519.61 (COO-) and 1348.51 (*β* (CH)_C=C_ + *β*OH) medium signals 1072.23 [*β* (CH)], 671.50 (COO^–^), 665.02 (CC), 455.31 (CC) and 442.72(CH) were observed. Based on a comparison of ESI-MS and FT-IR with the reported values in the literature (Ruelas et al. 2006; Swislocka et al. 2012; Umashankar et al. 2014, 2015; Akdemir et al. 2017), the bioactive compound was predicted to be *p*-Coumaric acid (C_9_H_8_O_3_) (PC) with a molecular weight of 164.04 Da (Figure 1).

The anti-bacterial and anti-yeast activities of EA and PC were determined qualitatively and quantitatively by measuring ZOIs and MICs against three Gram-positive bacteria, five Gram-negative bacteria and two yeast species (Table 1). For both EA and PC, the ZOIs and MICs ranged from 12.5 to 24.5 mm and from 7.8 to 125 µg/mL, respectively. Among the bacteria tested, the most susceptible bacterium against EA and PC was *S. faecalis* (MICs 15.6 and 7.8 µg/mL, respectively) and the most resistant bacterium was *P. aeruginosa* (MICs 125 and 62.5 µg/mL), respectively. Further, EA and PC also showed strong anti-yeast activity against *C. albicans* and *C. neoformans* with the ZOIs and MICs ranging from 16.6 to 23.3 mm and from 7.8 to 125 µg/L, respectively. The anti-microbial activities of PC were comparable with the standard drugs, erythromycin and itraconazole.

**Table 1.** Anti-bacterial and anti-yeast activities of EA, PC, erythromycin and itraconazole against some food-borne human pathogenic bacteria and yeast.

	EA		PC		Erythromycin/Itraconazole
Microbial isolates	ZOI	MIC	ZOI	MIC	ZOI
*E. coli*	13.5 ± 0.6	125	16.8 ± 1.0	62.5	10.1 ± 0.3
*K. pneumoniae*	17.2 ± 1.2	31.2	21.6 ± 1.3	15.6	15.5 ± 0.7
*P. vulgaris*	16.8 ± 0.8	62.5	19.6 ± 0.9	15.6	8.5 ± 0.4
*P. aeruginosa*	12.9 ± 0.6	125	16.5 ± 0.7	62.5	11.8 ± 0.6
*S. typhi*	17.9 ± 0.9	31.2	20.5 ± 0.8	31.2	19.5 ± 0.6
*S. aureus*	18.5 ± 1.2	31.2	21.4 ± 0.9	15.6	17.2 ± 0.5
*S. faecalis*	21.5 ± 1.4	15.6	24.5 ± 1.3	07.8	19.6 ± 0.7
*C. albicans*	16.6 ± 0.8	125	21.6 ± 1.1	31.2	13.5 ± 0.6
*C. neoformans*	20.7 ± 1.0	62.5	23.3 ± 1.0	07.8	14.8 ± 0.4

The data given are the means of four replicates ± standard error (*p* ≤ 0.05). The ZOIs values of EA (150 µg/disc) and PC (100 µg/disc) expressed in mm, and MICs values in µg/mL. Erythromycin (15 mcg/disc) served as positive control for bacteria and Itraconazole (10 mcg/disc) for yeast.

The EA and PC showed significant anti-fungal activities against 15 different seed-borne plant pathogenic fungi with %MI and MICs values ranging from 23.6 to 82.6 mm and from 7.8 to 250 µg/mL, respectively (Table 2). Among the fungi tested, the most susceptible fungus was *F. udum* (%MI 68.5% and 82.6%) followed by *C. tetramera* (%MI 63.2% and 80.4%), whereas *P. expansum* (%MI 23.6% and 54.6%) and *A. flavus* (%MI 28.9% and 58.4%) were recorded as the most resistant organisms. On a comparative evaluation of EA and PC with indofil Z-78, the %MI values of EA and PC were comparable with indofil Z-78.

**Table 2.** Anti-fungal activity of EA, PC, and indofil Z-78 against different field and storage fungi.

	EA		PC		Indofil Z-78
Fungal species	%MI	MIC	%MI	MIC	%MI
*A. brassicicola*	57.4 ± 2.8	31.2	73.4 ± 1.4	15.6	70.6 ± 0.8
*A. geophila*	51.5 ± 2.2	62.5	70.6 ± 1.9	31.2	67.4 ± 0.6
*A. flavus†*	28.9 ± 1.4	250	58.4 ± 1.0	125	51.5 ± 1.1
*A. fumigatus*	48.5 ± 2.1	125	65.6 ± 0.9	62.5	61.2 ± 0.9
*A. ochraceous*	50.6 ± 0.6	62.5	69.8 ± 0.8	31.2	58.6 ± 0.5
*A. tamarii*	28.5 ± 0.3	250	56.6 ± 0.6	125	52.6 ± 0.8
*A. terreus*	49.5 ± 1.0	125	66.8 ± 0.8	62.5	64.6 ± 1.2
*C. tetramera*	63.2 ± 2.3	31.2	80.4 ± 1.7	15.6	79.8 ± 1.0
*F. equiseti*	58.1 ± 1.5	31.2	82.2 ± 1.2	15.6	80.2 ± 1.
*F. lateritium*	51.8 ± 1.2	62.5	73.5 ± 0.7	31.2	68.4 ± 0.7
*F. oxysporum*	41.8 ± 1.0	125	75.8 ± 1.5	62.5	70.3 ± 0.7
*F. udum*	68.5 ± 1.3	15.6	82.6 ± 1.2	07.8	82.4 ± 1.5
*F. verticillioides‡*	59.8 ± 0.9	62.5	71.6 ± 1.0	31.2	65.6 ± 0.9
*P. citrinum*	28.5 ± 0.6	125	59.8 ± 0.8	125	56.8 ± 0.5
*P. expansum*	23.6 ± 0.8	250	54.6 ± 1.3	125	51.4 ± 0.7

The data given are the means of three replicates ± standard error (*p* ≤ 0.05). The %MIs values of EA (150 µg/mL) and PC (100 µg/mL) are expressed in percentage, and MICs values are expressed in µg/mL. Indofil Z-78 (2 mg/mL) served as a positive control. †Aflatoxin B_1_ producing strain and ‡Fumonisin B_1_ producing strain.

**Figure 2.**
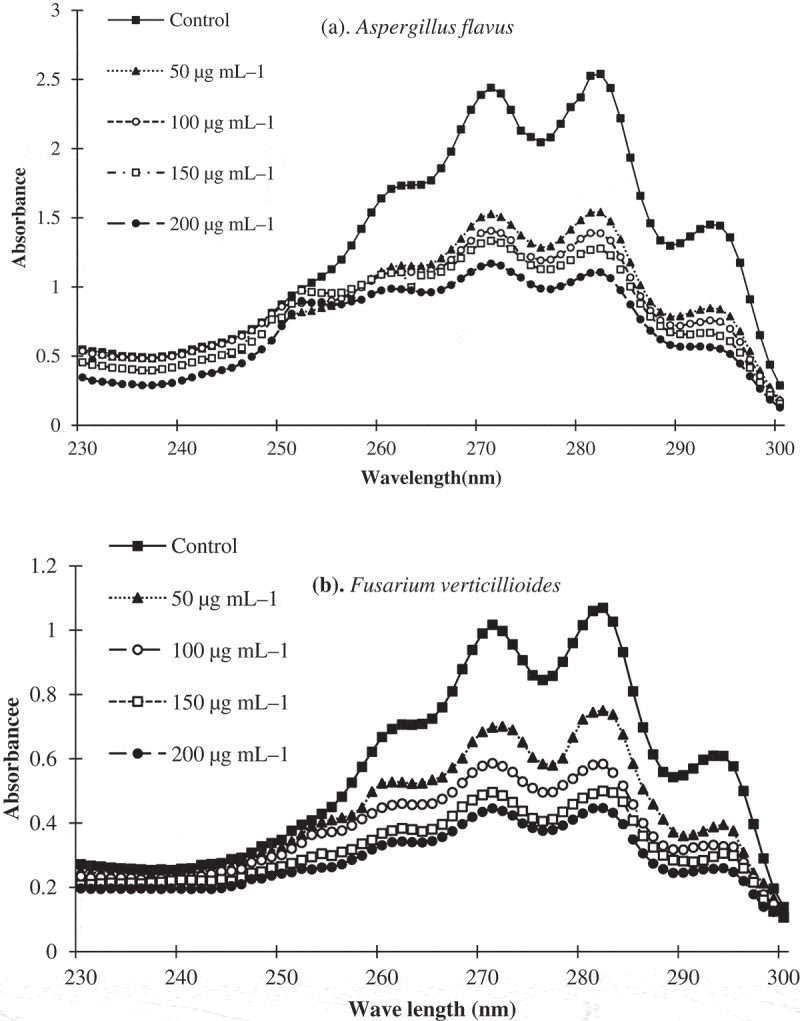
Inhibitory effect of p-Coumaric acid on the synthesis of ergosterol from *A. flavus* (a) and *F. verticillioides* (b).

The AFB_1_ production from *A. flavus* and FB_1_ production from *F. verticillioides* were significantly inhibited by EA and PC (Table 3). The control sets showed the presence of the highest amount of AFB_1_ and FB_1_ but the *in vitro* production of these toxins was completely inhibited by EA and PC at 400 µg/mL. In the viable maize model assay, both EA and PC significantly protected maize seeds from a wide range of fungal infestation and also inhibited AFB_1_ and FB_1_ synthesis from *A. flavus* and *F. verticillioides* at 400 µg g*^–^*^1^. Based on the values of percent incidences (PI), *Alternaria* (PI 38.7%), *Aspergillus* (PI 82.6%), *Cladosporium* (PI 21.5%), *Curvularia* (PI 26.7%), *Fusarium* (PI 71.6%), *Penicillium* (PI 59.6%), *Rhizopus* (PI 18.6%) and *Trichoderma* (PI 15.0%) were recorded as the dominant fungi in the control seed samples, but the emergence of these fungal species viz., *Alternaria, Aspergillus, Cladosporium, Curvularia, Fusarium, Penicillium, Rhizopus* and *Trichoderma* were significantly inhibited by EA and PC with PI values 10%, 35%, 0%, 9%, 15%, 32%, 0% and 2% in case of EA, and 0%, 10%, 0%, 0%, 0%, 14%, 0% and 0% in case of PC, respectively. Compared to the seedling-vigour index of the control samples (2245), the seedling-vigour index in EA (2472) and PC (2580) treated maize samples was significantly increased at 400 µg kg*^–^*^1^ treatment. The ergosterol synthesis from *A. flavus* and *F. verticillioides* was significantly inhibited by PC (Figure 2) and the percent reduction in the ergosterol in comparison with the control was 24.56, 37.56, 46.84 and 58.45%, and 46.89, 58.34, 68.95 and 76.93% at 50, 100, 150 and 200 µg/mL, respectively.

**Table 3.** Efficacy of EA and PC on AFB_1_ production from *A. flavus* and FB_1_ production *F. verticillioides.*

	AFB_1_ production from *A. flavus*				FB_1_ production from *F. verticillioides*			
	*In vitro*		*In vivo*		*In vitro*		*In vivo*	
Extract concentrations (EA/PC)	EA	PC	EA	PC	EA	PC	EA	PC
Control	1455.6 ± 8.9	1455.6 ± 8.9	1586.8 ± 9.7	1586.8 ± 9.7	82.9 ± 3.8	82.9 ± 3.8	50.5 ± 3.7	50.5 ± 3.7
50	1005.2 ± 7.6	768.4 ± 7.6	1285.6 ± 8.8	1145 ± 4.7	41.6 ± 2.5	22.7 ± 1.8	41.6 ± 2.5	33.7 ± 2.1
100	614.6 ± 3.9	143 ± 1.8	895.5 ± 4.5	685 ± 4.5	10.8 ± 0.7	0.0 ± 0.0	25.4 ± 2.2	14.4 ± 0.8
200	135.8 ± 2.6	0.0 ± 0.0	564.4 ± 2.7	145 ± 2.7	0.0 ± 0.0	0.0 ± 0.0	12.9 ± 1.7	0.0 ± 0.0
400	0.0 ± 0.0	0.0 ± 0.0	195.7 ± 1.6	0.0 ± 0.0	0.0 ± 0.0	0.0 ± 0.0	0.0 ± 0.0	0.0 ± 0.0

The data given are the means of three replicates ± standard error (*p* ≤ 0.05). The values of AFB_1_ production are expressed in µg/mL under *in vitro* and µg/g under *in vivo*. The values of FB_1_ production are expressed in mg/mL under *in vitro* and mg/g under *in vivo*.

## Discussion

The present study confirms that both EA and PC show broad-spectrum anti-microbial activity against bacteria, yeast and fungi. Among the bacteria tested, the Gram-positive bacteria are more susceptible than the Gram-negative ones. Similarly, the field fungi were more susceptible than the storage fungi. The obtained results confirm the potential of EA and PC in the management of diseases caused by Gram-positive bacteria and field fungi. The broth dilution method was employed for the determination of MICs of EA and PC because this method facilitates better interaction between extracts and micro-organisms than the diffusion techniques (Prakash et al. 2014). The values of MIC of EA and PC were comparable or greater than the synthetic agents erythromycin, itraconazole, and Indofil Z-78, indicating possible exploitation of endophytic *A. alternata* for the production of large-scale chemotherapeutic and anti-mycotoxigenic agents. Maize is a nutritionally important staple food for human but it is susceptible to fungal infestation either in the field before harvest or at a post-harvest stage such as storage and processing. The common fungal flora reported in maize samples worldwide includes the species of *Alternaria, Aspergillus, Chaetomium, Cladosporium, Curvularia, Drechslera, Fusarium Penicillium, Phoma* and *Trichoderma* (Soares et al. 2013; Mohana et al. 2016). In the *in vivo* studies, the conditions used in the viable maize model were similar to the conditions prevalent during the storage. The obtained results confirm that both EA and PC were effective in suppressing a wide number of seed-borne fungi. The inhibition of mycotoxin production suggests their possible use as protective agents during storage of food grains and feed-stuffs. Ergosterol is a fungal-specific sterol component in the cell membrane and also one of the potential target sites for the action of many anti-fungal drugs (Tian et al. 2012). The depletion of ergosterol alters the activity of several membrane-bound enzymes related to nutrient transport and chitin synthesis. The present study confirms that the ergosterol synthesis from *A. flavus* was strongly inhibited by PC, which indicates ergosterol bound to the plasma membrane is a possible target for the anti-fungal action of PC.

A high number of bioactive secondary metabolites have been isolated from *Alternaria* (Gu et al. 2009; Lou et al. 2013; Bhagat et al. 2016). The extracts of endophytic *A. alternata* and *A. brassicicola* have shown anti-microbial activity against *B. subtilis, E. coli, Mycobacterium tuberculosis, P. fluorescens* and *C. albicans* (Qiao et al. 2007; Fernades et al. 2009; Sonaimuthu et al. 2011; Srivastava et al. 2015). Bhagat et al. (2016) reported that the bioactive compound, altenuene, isolated from *A. alternata* an endophytic fungus of *Catharanthus roseus* shows significant antioxidant and insecticidal activity. Umashankar et al. (2014; 2015) reported that the bioactive compound *p*-Coumaric acid isolated from endophytic *Alternaria* species shows anti-cancer activity against HeLa cancer cell line. As per best of our knowledge, there is no report on the anti-microbial activities of EA and PC from *A. alternata* isolated from *C. roseus* against a wide-range of food-borne bacteria, yeast and fungi, and further this is also the first report on the anti-aflatoxigenic and anti-fumonisinic properties of EA and PC. Hence, EA and PC could be explored as anti-microbial agents for the protection of food and feedstuff against microbial deterioration and mycotoxins contamination.
